# Effects of Upper and Lower Limb Plyometric Training Program on Components of Physical Performance in Young Female Handball Players

**DOI:** 10.3389/fphys.2020.01028

**Published:** 2020-08-18

**Authors:** Mehrez Hammami, Nawel Gaamouri, Katsuhiko Suzuki, Roy J. Shephard, Mohamed Souhaiel Chelly

**Affiliations:** ^1^Research Unit (UR17JS01), Sport Performance, Health & Society, Higher Institute of Sport and Physical Education of Ksar Saïd, Manouba University, Tunis, Tunisia; ^2^Higher Institute of Sport and Physical Education of Ksar Saïd, Manouba University, Tunis, Tunisia; ^3^Faculty of Sport Sciences, Waseda University, Tokorozawa, Japan; ^4^Faculty of Kinesiology and Physical Education, University of Toronto, Toronto, ON, Canada

**Keywords:** change-of-direction, stork test, best time, mean time, push up jump, hurdle jump

## Abstract

**Purpose:**

This study examined the effects of a 10-week combined upper and lower limb plyometric training (ULLPT) programs on components of physical performance in young female handball players.

**Methods:**

Participants aged 15.8 ± 0.2 years were randomly assigned between the experimental (EG; *n* = 17) and control (CG; *n* = 17) groups. Two-way analyses of performance (group × time) assessed changes in handgrip force, back extensor strength; medicine ball throwing, 30-m sprint times, change of direction (CoD) [Modified Illinois test (Illinois-MT)], four jumping tests [squat jump (SJ), countermovement jump (CMJ), CMJ with arms (CMJA) and 5 jump test (5JT), static and dynamic balance, and repeated sprint T-test scores (RSTT)].

**Results:**

After 10 weeks of plyometric training (two sessions per week), group × time interactions showed significant changes in EG relative to CG in right and left handgrip force, back extensor strength and medicine ball throwing [*p* < 0.001, *d* = 1.51 (large); *p* < 0.0001, *d* = 0.85 (large); *p* < 0.001, *d* = 0.90 (large); *p* < 0.0001, *d* = 0.52 (medium), respectively]. Group × time interactions also showed improvements of EG relative to CG in sprint times [5 m (*p* = 0.02, *d* = 0.80 (large)); 10 m (*p* < 0.0001, *d* = 1.00 (large)); 20 m (*p* = 0.02, *d* = 1.41 (large)); and 30 m (*p* = 0.02, *d* = 2.60 (large))], CoD [Illinois-MT (*p* < 0.001, *d* = 1.58 (large))] and jumping [(SJ, CMJ, CMJA, and 5JT, *p* = 0.001, *d* = 0.87 (large); *p* < 0.001, *d* = 1.17 (large); *p* < 0.001, *d* = 1.15 (large); and *p* = 0.006, *d* = 0.71 (medium)) respectively]. Further, all RSTT scores (best time, mean time, total time, and fatigue index) improved significantly in the experimental group, with group × time interactions varying between *p* < 0.001 and *p* = 0.049 (*d* value large to medium). However, balance did not differ significantly between EG and CG.

**Conclusion:**

We conclude that 10 weeks of ULLPT improved many measures of physical performance in young female handball players.

## Introduction

The muscular power and strength of both the upper and the lower limbs are key contributors to performance in handball (Michalsik and Aagaard, 2015; Wagner et al., 2018), and plyometric training with quick and powerful multi-joint movements like jumping, hopping, and skipping is frequently used in handball to improve these aspects of a player’s physical fitness (Chaabene et al., 2019; Hammami et al., 2019; Prieske et al., 2019). The response to such training depends on subject characteristics including age, sex, and initial training level, as well as program characteristics (type of training, exercise surface, and the rest interval between sets and training sessions) (Markovic and Mikulic, 2010; Ramirez-Campillo et al., 2018; Hammami et al., 2019). However, there is little information concerning its impact in young female handball players, especially in terms of upper limb training, although one might anticipate a particularly large response in those with minimal previous experience of plyometric training (Rubley et al., 2011). Hammami et al. (2019) found increases in both upper limb (handgrip force, back extensor strength, and medicine ball throwing) and lower limb [sprinting, change of direction (CoD), and jumping] performance after 9 weeks of combined upper and lower limb plyometric training (ULLPT) in U14 female handball players, and Chaabene et al. (2019) also reported increases in fitness measures after 8 weeks of plyometric training in young female handball players. On the other hand, Meszler and Vaczi (2019) found no significant changes in T agility test scores, balance, hamstring strength or H:Q ratio after 7 weeks of plyometric training in female basketball players aged less than 17 years. Haj-Sassi et al. (2011) concluded that players of a number of field and court sports performed many intermittent forward, backward, and lateral high speed movements during competitions. The ability to change direction is thus an important skill in many field and court sports. In team handball, it is essential to react quickly and perform powerful changes in direction, while moving quickly over short distances (Michalsik et al., 2014). In a typical handball game, there are 279 changes-of-direction, in response to visual or auditory cues (Cuesta, 1991). Players must change direction with a minimal loss of speed, balance, and motor control, and make short, maximal efforts with only brief recovery periods. Moreover, the ability to perform repeated sprints and changes of direction is regarded by many coaches and researchers as an important predictor of superior performance in intermittent and team sports (Wong Del et al., 2012).

No previous study has examined the impact of combined ULLPT on the ability of young females to change direction repeatedly. Thus, this study investigated the effects of a progressive in-season ULLPT training program on selected components of physical performance (strength, sprinting, jumping, CoD, balance, and repeated CoD) in young female handball players. We hypothesized that the inclusion of ULLPT into the regular training routine of young female handball players would generate larger improvements in physical performance than those obtained by standard handball training alone.

## Materials and Methods

### Ethical Approval

All procedures were approved by the local ethical committee for the use of human participants. The study was conducted in accordance with the latest version of the Declaration of Helsinki. Written informed parental consent (for those <17 years) and participants’ consent were obtained prior to commencing the study. All participants and their parents/legal representatives were fully informed about the experimental protocol and its potential risks and benefits.

### Participants

The G power 3.0.10 program was used to calculate the minimal sample size needed in our study, with Z_1–β_ = 1.03 (power = 85%) and *Z_α/2_* = 1.96 (alpha = 5%). The study of Chaabene et al. (2019) showed the mean ± SD of 5-m sprint performance as 1.24 ± 0.12 (s) in the experimental group vs 1.17 ± 0.06 (s) in the control group (CG), and considering a ratio of one control for every case, there was a need for 15 experimental and 15 control subjects (Faul et al., 2007). Thirty-four young female handball players from two different clubs were randomly assigned between a plyometric training group (EG; *n* = 17; age = 15.8 ± 0.2 years; body mass = 64.2 ± 3.3 kg; height = 1.66 ± 0.03 m; body fat = 25.2 ± 3.8%; maturity-offset = 3.0 ± 0.5 years) and a CG who maintained their standard in-season regimen (*n* = 17; age = 15.8 ± 0.2 years; body mass = 63.0 ± 3.8 kg; height = 1.67 ± 0.04 m; body fat = 26.0 ± 3.3%; maturity-offset = 3.0 ± 0.4 years). All players were competing at the national level and were therefore classified as elite athletes. Their mean experience of handball competition was 5 years. They had 3 years’ experience of plyometric training. All had already achieved a good overall physical preparation at the beginning of the season (a preliminary 6-week period of six training sessions per week). This preliminary phase was divided into two parts. During the first 3 weeks, a resistance training program aimed to improve muscular power and muscle volume by light loads (40–60%, one repetition maximum). The second 3-week period was devoted to improving muscular strength with higher loads (70–85%, one repetition maximum), supplemented by participation in friendly matches each weekend. Subjects continued to engage in five sessions per week of training during September, when the championship season began. The experimental intervention of biweekly ULLPT was undertaken during the second phase of the national championships (January to March). All participants had previously engaged in five to six training sessions per week (90-to-120 min each session). However, for 10 weeks, the EG replaced some of their handball-specific drills with a plyometric training program, although the overall training volume remained comparable for the two groups. It was decided that if any athlete missed more than 10% of the total training sessions and/or more than two consecutive sessions would be excluded from the study.

### Experimental Design

**FIGURE 1:**
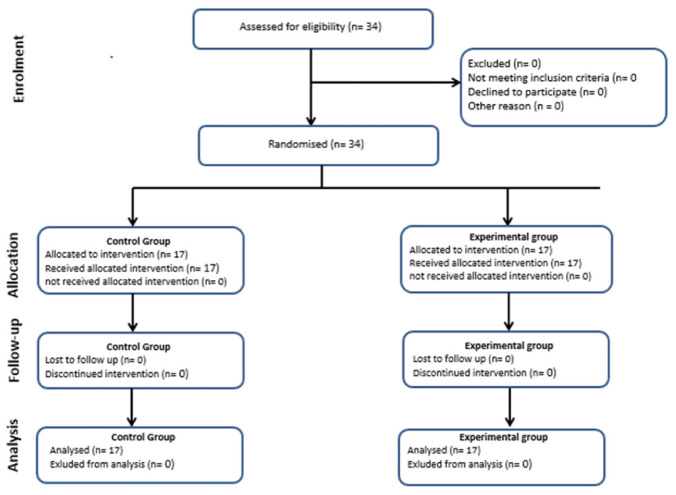
The diagram (The CONSORT: Consolidated Standards of Reporting Trials) includes detailed information on the interventions received.

The study was designed to test the effects of a 10-week ULLPT program on selected fitness measures in young female handball players. Figure 1 presents a revised CONSORT diagram of the levels of reporting and explaining participant flow. The training intervention was conducted in-season during 2018–2019. In the week prior to the intervention, two 80- to 90-min sessions familiarized players with all test procedures. Measurements were made in a fixed order over 4 days, immediately before and 4 days after the last plyometric training session. Subjects did not participate in any exhausting exercise for 24 h before testing, and no food or caffeine-containing drinks were taken for 2 h before testing. A standardized warm-up (10–20 min of low- to moderate-intensity aerobic exercise and dynamic stretching) preceded all tests. On the first test day, participants made a 30-m sprint, followed by the modified Illinois change-of-direction test; three trials were allowed for each test (separated by 6–8 min of recovery) and the best performances as determined by paired photocells (Microgate, Bolzano, Italy) were noted. The second day was devoted to jumping [squat jump (SJ), countermovement jump (CMJ), CMJ with arms (CMJA), and horizontal 5-jump tests (5JT)] followed by dominant and non-dominant handgrip force assessments. On the third day, anthropometric measurements were followed by determinations of back extensor strength (three trials separated by at least 2 min of recovery) and medicine ball throw tests (two trials separated by 5 min of recovery), with the best attempts noted for further analyses. On the fourth day, the stork test, *Y* balance test and repeated sprint *T*-test (RSTT) were completed.

### Testing Procedures

#### 30-m Sprint Performance

Players started from a standing position, with the front foot 0.2 m from the first photocell beam and ran 30 m, with times recorded over distances of 5, 10, 20, and 30 m.

#### Modified Illinois Change-of-Direction Test (Illinois-MT)

Four cones formed the change-of-direction area for the modified Illinois test. On command, players sprinted 5 m, turned and ran back to the starting line, then, swerving in and out of the four markers, and completed two 5-m sprints (Hachana et al., 2014). No advice was given as to the most effective technique, but players were instructed to complete the test as quickly as possible without cutting over markers. Five minutes of rest was allowed between each of the three trials, and the fastest performance was used in subsequent analyses.

#### Vertical Jump

Jump height was assessed using an infrared photoelectric measurement device connected to a digital computer (Optojump System; Bolzano, Italy) that measured contact and flight times and the height of jump with a precision of 1/1,000 s. Participants begun the SJ at a knee angle of ∼90° (self-controlled, using a mirror), avoiding any downward movement, and pushed upward, keeping their legs straight throughout. The CMJ began from an upright position; a rapid downward movement to a knee angle of ∼90° (again self-controlled, using a mirror) accompanied the beginning of the push-off. During the CMJA, the hands were used freely while jumping. One minute of rest was allowed between three trials of each test, and the highest jump of each type was used in subsequent analyses.

#### Five Jump Test (5JT)

The test was performed as previously described (Hammami et al., 2019). From an upright standing position with both feet flat on the ground, participants tried to cover as much distance as possible with five forward jumps, alternating left- and right-leg ground contacts. Participants were allowed three maximal trials, with 3 min of rest between efforts, and the best performance was used for analyses.

#### Handgrip Force

A hand dynamometer (Takei, Tokyo, Japan) was held with the arm at right angles and the elbows at the side of the body. The instrument was adjusted so that its base rested on the first metacarpal and the handle rested on the middle of the participant’s four fingers. A maximal isometric effort was maintained for 5 s, without ancillary body movements. Two trials were made with each hand, with 1 min of rest between trials, and the highest readings were used in subsequent analyses.

#### Anthropometry

Anthropometric measurements included standing and sitting body height (stadiometer accuracy of 0.1 cm; Holtain, Crosswell, Crymych, Pembs, United Kingdom) and body mass (0.1 kg; Tanita BF683W scales, Munich, Germany). The overall percentage of body fat was estimated from the biceps, triceps, subscapular, and suprailiac skinfolds, using the equations of Durnin and Womersley (1974) for children and adolescent females:

%Body fat=(495/D) − 450

where *D* = 1.1369 − 0.0598 (Log sum of 4 skinfolds).

Maturity offset status was calculated from peak height velocity (Mirwald et al., 2002):

Maturity offset = −9.38 + (0.000188 × leg length × sitting height) + (0.0022 × age × leg length) + (0.00584 × age × sitting height) + (0.0769 × weight/height ratio).

#### Back Extensor Strength

Maximal isometric back extensor strength was measured using a back extensor dynamometer (Takei, Tokyo, Japan) (Hannibal et al., 2006). Participants stood on the dynamometer, with their feet a shoulder width apart and gripped the handle bar positioned across the patellae. The chain length was adjusted so that initially the legs were held straight and the back was flexed to 30°, as guided by wall markings. Participants then stood upright without bending their knees, pulling upward as strongly as possible.

#### Medicine Ball Throw

The test was performed using a 3-kg rubber medicine ball with 21.5-cm diameter (Tigar, Pirot, Serbia) powdered with magnesium carbonate. A familiarization session included a brief description of the optimal technique (Gillespie and Keenum, 1987). The seated player grasped the medicine ball with both hands, and on signal forcefully pushed the ball from the chest. The score was measured from the front of the sitting line to the powder-marked spot where the ball landed.

#### Stork Balance Test

Subjects stood on their dominant leg with their opposite foot resting against the inside of the supporting knee and both hands on their hips (Negra et al., 2017). On signal, they raised their heel; the test was terminated when the heel touched the ground or the foot moved away from the patella.

#### Dynamic Balance Test

Dynamic balance was assessed for both right and left legs, using the *Y*-balance test (Negra et al., 2017). Supine leg lengths were first determined from the anterior superior iliac spine to the most distal aspect of the medial malleolus. Subjects then stood barefoot and single-legged, with the tip of their great toe at the center of the grid, and reached in anterior, postero-medial and postero-lateral directions, marked on the floor by tape. The posterior lines extended at an angle of 135° from the anterior line. Trials were repeated if the participant (1) did not touch the required line with the reaching foot while maintaining weight-bearing on the stance leg, (2) lifted the stance foot from the center of the grid, (3) lost balance, (4) did not maintain start and return positions for one full second, or (5) touched the reaching foot to gain support. The maximal reach was measured in each direction, and a composite score calculated as [(maximum anterior + maximum postero-medial + maximum postero-lateral reach distance)/(leg length × 3) × 100] (Negra et al., 2017). Three trials were conducted in each direction, with two-minute rest intervals.

#### Repeated Sprint *T*-Test (RSTT)

This test offers a reliable and valid measurement (Pauole et al., 2000) of the ability to change directions rapidly, simulating a game with short, intense efforts, recovery periods and multi-directional displacements. Seven executions of the RSTT were made, with subjects walking back slowly to the next start point during 25-s recovery intervals. Measures included best time (RSTT-BT), mean time (RSTT-MT), total time (RSTT-TT) and a fatigue index (RSTT-FI) calculated as (Fitzsimons et al., 1993):

FI=[(Total time/(Best time × 7)) × 100]−100

### Plyometric Training Program

The intervention consisted of a progressive 10-week ULLPT program, based on the players’ previous training records and research results (Hammami et al., 2019). It was completed during the mid-portion of the competitive season (from January to March) (Table 1). Biweekly plyometric sessions (Tuesdays and Thursdays) were performed immediately after the warm-up program, replacing some low-intensity technical-tactical handball drills. The intervention included push-up exercises for the upper limbs, and hurdling, lateral hurdling, and hurdle jumping (jumping with 180° rotation) exercises for the lower limbs. Exercises for the upper limbs were immediately followed by lower-limb exercises (i.e., 6 to 10 repetitions of dynamic push-ups + 6 to 8 repetitions of lower limb jumps), with no intervening rest periods (Table 1). The sequence of plyometric exercises for the upper and lower limbs lasted ∼10 s (Stojanovic et al., 2017). Recovery between sets was for 30 s. All plyometrics in general (i.e., upper and lower limb exercises) were performed with maximal effort, minimizing contact time in each repetition, and no resting was allowed between jumps. With the exception of competitive and friendly matches, the ULLPT replacement activity accounted for <10% of the total training load. During the intervention, the CG followed their regular handball training (i.e., mainly technical-tactical drills, small sided and simulated games, and injury prevention drills). The overall training load was measured and comparable between groups using a rating of perceived exertion (Helms et al., 2016).

**TABLE 1:** Plyometric training program.

	Weeks 1–2	Weeks 3–4	Weeks 5–6	Weeks 7–8	Weeks 9–10
	S × R	S × R	S × R	S × R	S × R
**Upper limb**					
Push-up	10 × 6	10 × 6	10 × 6	10 × 6	10 × 6
Contacts number	60	60	60	60	60
	H × S × R	H × S × R	H × S × R	H × S × R	H × S × R
**Lower limb**					
Hurdle jump	0.3 m × 2 × 6	0.3 m × 3 × 6	0.35 m × 2 × 6	0.35 m × 3 × 6	0.4 m × 2 × 6
Lateral hurdle jump	0.3 m × 2 × 6	0.3 m × 3 × 6	0.35 m × 2 × 6	0.35 m × 3 × 6	0.4 m × 2 × 6
Stretched leg jump	0.25 m × 2 × 6	0.25 m × 3 × 6	0.30 m × 2 × 6	0.30 m × 3 × 6	0.35 m × 2 × 6
Hurdle jump (jump with 180° rotation)	0.25 m × 2 × 6	0.25 m × 3 × 6	0.30 m × 2 × 6	0.30 m × 3 × 6	0.35 m × 2 × 6
Horizontal jump	1.1 m × 2 × 6	1.1 m × 3 × 6	1.2 m × 2 × 6	1.2 m × 2 × 6	1.3 m × 2 × 6
Contact number	60	90	60	90	60

*H, height; S, sets; R, reps.*

### Statistics

Statistical analyses were carried out using the SPSS 20 program for Windows (SPSS, Inc., Armonk, NY: IBM Corp). Normality of all variables was tested using the Kolmogorov–Smirnov test procedure. A few results (Handgrip, *Y* Balance test and Stork balance test) showed some skewing of data, and both means and medians were reported for these measurements. Data are presented as mean (SD), and as median values for skewed variables. Between-group differences at baseline were examined using independent *t*-tests, and the effect of the intervention was determined by two-way analyses of variance (Experimental vs Control and Test vs Retest). To evaluate within-group pre-to-post performance changes, paired sample *t*-tests were applied. Effect sizes were calculated by converting partial eta squared values to Cohen’s *d* [classified as small (0.00 ≤ *d* ≤ 0.49), medium (0.50 ≤ *d* ≤ 0.79), and large (*d* ≥ 0.80)] (Cohen, 1988). Training-related effects were assessed by two-way analyses of variance (group × time). The criterion for statistical significance was set at *p* < 0.05, whether a positive or a negative difference was seen (i.e., a two-tailed test was adopted). The reliabilities of all dependent variables were assessed by calculating intra-class correlation coefficients (two-way mixed) and coefficients of variation.

## Results

No athletes missed more than 10% of the total training sessions and/or more than two consecutive sessions, so it was not necessary to exclude any participants from the study.

### Reliability of the Tests

Test-retest reliabilities were generally above the accepted threshold, with intra-class correlation coefficients ranging from 0.63 to 0.98, and coefficients of variation of 1.4 to 58.4%. However, the stork left test had a poor reliability (ICC = 0.637/CV = 58.4) (Table 2).

**TABLE 2:** Reliability and variability of performance tests.

	ICC	95% CI	CV
5 m	0.975	0.950–0.987	4.7
10 m	0.957	0.915–0.979	2.6
20 m	0.953	0.906–0.976	1.4
30 m	0.982	0.963–0.991	9.0
Illinois-MT	0.909	0.812–0.954	2.1
SJ	0.910	0.819–0.955	8.2
CMJ	0.978	0.956–0.989	7.5
CMJA	0.929	0.857–0.964	7.3
5JT	0.971	0.942–0.986	8.0
Handgrip right	0.896	0.791–0.948	7.3
Handgrip left	0.869	0.738–0.935	8.2
Back extensor	0.867	0.735–0.934	10.1
Medicine ball throw	0.978	0.955–0.989	18.1
Stork right	0.853	0.705–0.926	46.5
Stork left	0.637	0.274–0.819	58.4
*Y* balance test (RL/L)	0.977	0.54–0.988	11.0
*Y* balance test (RL/B)	0.894	0.788–0.947	9.8
*Y* balance test (RL/R)	0.942	0.883–0.971	19
*Y* balance test (LL/R)	0.982	0.963–0.991	9.0
*Y* balance test (LL/B)	0.942	0.883–0.971	8.5
*Y* balance test (LL/L)	0.973	0.946–0.987	21.8

*CI, confidence intervals; CV, coefficient of variation; CMJ, counter-movement jump; CMJA, counter-movement jump; ICC, intraclass correlation coefficient; Illinois-MT, Illinois modified test; SJ, squat jump; B, background; L, left; R, right; LL, left leg; RL, right leg.*

### Between-Group Differences at Baseline

There were no significant initial intergroup differences for any of the dependent variables.

### Training-Related Effects

All data for both groups increased significantly over the 10-week intervention, with the exception of the Stork balance test (left leg), which remained unchanged for the CG (Tables 3, 4). The experimental group enhanced their upper limb performance scores: handgrip right (Δ = 26%, *p* < 0.0001, *d* = −4.10), handgrip left (Δ = 23%, *p* < 0.0001, *d* = −1.13), back extensor strength (Δ = 28%, *p* < 0.0001, *d* = −2.24), and medicine ball throw (Δ = 27.6%, *p* < 0.0001, *d* = −1.86), with significant group × time interactions [*p* < 0.001, *d* = 1.51 (large); *p* < 0.0001, *d* = 0.85 (large); *p* < 0.001, *d* = 0.90 (large); *p* < 0.0001, *d* = 0.52 (medium), respectively] (Table 4). The EG also enhanced their sprint performance over 5 m (Δ = 10%, *p* < 0.0001, *d* = 2.43), 10 m (Δ = 6.7%, *p* < 0.0001, *d* = 2.89), 20 m (Δ = 5.7%, *p* < 0.0001, *d* = 4.33), and 30 m (Δ = 7.8%, *p* < 0.0001, *d* = 6.10), with a group × time interaction [*p* = 0.02, *d* = 0.80 (large); *p* < 0.0001, *d* = 1.00 (large); *p* = 0.02, *d* = 1.41 (large); and *p* = 0.02, *d* = 2.60 (large), respectively]. Further, the EG improved their performance in the Illinois-MT (Δ = 7.4%, *p* < 0.0001, *d* = 12.40), with a group × time interaction [*p* < 0.001, *d* = 1.58 (large)] (Table 4). Jumping also improved significantly in the EG, with gains in the SJ (Δ = 18.2%, *p* < 0.0001, *d* = −2.47), CMJ (Δ = 21.1%, *p* < 0.0001, *d* = −3.09), CMJA (Δ = 20%, *p* < 0.0001, *d* = −2.82), and horizontal 5JT (Δ = 15.3%, *p* < 0.0001, *d* = −3.50), with group × time interactions at *p* = 0.001, *d* = 0.87 (large); *p* = 0.02, *d* = 1.17 (large); *p* < 0.001, *d* = 1.15 (large); and *p* = 0.006, *d* = 0.71 (medium), respectively. Again, all measures of RSTT performance increased significantly in the EG relative to the CG, with group × time interactions at *p* < 0.001, *d* = 1.57 (large); *p* < 0.001, *d* = 1.81 (large); *p* < 0.001, *d* = 1.81 (large); and *p* = 0.049, *d* = 0.50 (medium), in RSTT-BT, RSTT-MT, RSTT-TT, and RSTT-FI, respectively (Table 4). However, group × time interactions showed no significant inter-group differences in either static or dynamic balance (Table 4).

**TABLE 3:** Upper-limb performance in experimental and control groups before and afterthe 10-week intervention.

	Experimental group (*n* = 17)					Control group (*n* = 17)					Anova group × time interaction	
			
	Pre	Post	% Δ change	Paired *t* test		Pre	Post	% Δ change	Paired *t* test		*p*	*d* (cohen)
								
				*p*	*d* (cohen)				*p*	*d* (cohen)		
Handgrip right (N)	233 ± 14	295 ± 17	26.3 ± 6.1	<0.001	4.10	231 ± 20	240 ± 19	4.1 ± 1.7	<0.001	0.48	<0.001	1.51
Handgrip left (N)	227 ± 22	279 ± 38	23.0 ± 6.2	<0.001	1.73	226 ± 14	239 ± 13	5.8 ± 2.7	<0.001	0.99	0.001	0.85
		(267)^a^										
Back extensor (N)	765 ± 97	980 ± 101	28.4 ± 4.5	<0.001	2.24	792 ± 56	861 ± 70	8.8 ± 6.0	<0.001	1.12	0.001	0.90
Medicine ball throw (m)	3.1 ± 0.5	4.0 ± 0.5	27.6 ± 9.6	<0.001	1.86	3.2 ± 0.6	3.4 ± 0.6	8.2 ± 7.8	<0.001	0.34	0.041	0.52

*^*a*^Median reported for rightward skewing data.*

**TABLE 4:** Lower-limb performance in experimental and control groups before and after the 10-week intervention.

	Experimental group (*n* = 17)					Control group (*n* = 17)					Anova group × time interaction	
			
	Pre	Post	%Δ change	Paired *t* test		Pre	Post	%Δ change	Paired *t* test		*p*	*d* (cohen)
								
				*p*	*d* (cohen)				*p*	*d* (cohen)		
**Sprint**												
5 m (s)	1.25 ± 0.06	1.12 ± 0.05	10.0 ± 1.9	<0.001	2.43	1.28 ± 0.05	1.24 ± 0.05	3.3 ± 1.8	<0.001	0.82	0.002	0.80
10 m (s)	2.19 ± 0.05	2.05 ± 0.05	6.7 ± 1.5	<0.001	2.89	2.24 ± 0.05	2.20 ± 0.07	1.7 ± 2	0.002	2.37	<0.001	1.00
20 m (s)	3.77 ± 0.05	3.56 ± 0.05	5.7 ± 0.9	<0.001	4.33	3.75 ± 0.05	3.68 ± 0.07	1.8 ± 1	<0.001	1.19	<0.001	1.41
30 m (s)	4.64 ± 0.05	4.28 ± 0.07	7.8 ± 1.1	<0.001	6.10	5.54 ± 0.05	5.49 ± 0.07	1.0 ± 0.6	<0.001	0.85	<0.001	2.60
**Change of direction**												
Illinois-MT	13.07 ± 0.07	12.10 ± 0.09	7.4 ± 0.4	<0.001	12.40	13.11 ± 0.39	13 ± 0.39	0.8 ± 0.2	<0.001	0.29	<0.001	1.58
Jump												
SJ (cm)	22.4 ± 1.7	26.4 ± 1.9	18.2 ± 2.3	<0.001	2.47	22.8 ± 2.1	23.8 ± 1.6	4.5 ± 4.2	<0.001	0.55	0.001	0.87
CMJ (cm)	24.2 ± 1.6	29.3 ± 1.8	21.1 ± 3.3	<0.001	3.09	24.0 ± 2.0	24.9 ± 1.8	4.0 ± 3.3	<0.001	0.49	<0.001	1.17
CMJA (cm)	25.3 ± 1.6	30.4 ± 2.1	20.0 ± 2.8	<0.001	2.82	25.3 ± 2.1	25.8 ± 2.1	2.3 ± 1.1	<0.001	0.25	<0.001	1.15
5JT (m)	8.1 ± 0.3	9.3 ± 0.4	15.3 ± 1.4	<0.001	3.50	8.2 ± 1.5	8.5 ± 1.4	4.4 ± 3.3	<0.001	0.21	0.006	0.71
**RSTT**												
RSTT-BT (s)	12.61 ± 0.16	11.91 ± 0.18	5.5 ± 1.0	<0.001	4.24	12.61 ± 0.18	12.48 ± 0.23	1 ± 0.6	<0.001	0.65	<0.001	1.57
RSTT-MT (s)	12.93 ± 0.17	12.15 ± 0.16	6.1 ± 0.7	<0.001	4.87	12.90 ± 0.18	12.77 ± 0.23	1.0 ± 0.6	<0.001	0.65	<0.001	1.81
RSTT-TT (s)	90.5 ± 1.17	85.0 ± 1.15	6.1 ± 0.7	<0.001	4.89	90.30 ± 1.28	89.40 ± 1.58	1.0 ± 0.6	<0.001	0.65	<0.001	1.81
RSTT-FI (%)	2.52 ± 0.79(2.15)^a^	1.97 ± 0.87	19.3 ± 35.5	<0.001	0.68	2.30 ± 0.03	2.32 ± 0.04	1.0 ± 0.6	<0.001	0.58	0.049	0.50
***Y* Balance test**												
**Right support leg**												
RL/A (cm)	75 ± 7	79 ± 8	4.9 ± 2.3	<0.001	0.55	73 ± 9	79 ± 9	9.1 ± 6.7	<0.001	0.69	0.501	0.16
RL/BL (cm)	89 ± 8	92 ± 9	4.2 ± 1.3	<0.001	0.36	87 ± 9	92 ± 10	5.4 ± 3.8	<0.001	0.54	0.831	0.006
RL/BR (cm)	46 ± 9(44)^a^	52 ± 9(51)^a^	12.4 ± 5.1	<0.001	0.69	48 ± 7	54 ± 7	15.3 ± 19	0.001	0.88	0.841	0.006
**Left support leg**												
LL/A (cm)	78 ± 5	82 ± 6	4.2 ± 1.3	<0.001	0.75	78 ± 9	84 ± 7	8.8 ± 9.8	0.001	0.77	0.356	0.22
LL/BR (cm)	92 ± 8	99 ± 7	7.5 ± 2.3	<0.001	0.96	96 ± 8	99 ± 7	3.9 ± 2.9	<0.001	0.41	0.372	0.22
LL/BL (cm)	48 ± 12(43)^a^	51 ± 12	7.1 ± 3	<0.001	0.26	46 ± 8	50 ± 7	9.5 ± 7.6	<0.001	0.55	0.858	0.06
**Stork balance test**												
RL (s)	2.33 ± 1.16	3.37 ± 1.05	56.4 ± 39.8	<0.001	0.97	2.61 ± 1.16	3.92 ± 1.93(3.41)^a^	55.9 ± 51.2	0.003	0.85	0.685	0.10
LL (s)	2.56 ± 1.46(2.29)^a^	3.03 ± 1.46(2.76)^a^	22.2 ± 8.6	<0.001	0.33	2.58 ± 1.58(1.98)^a^	3.06 ± 1.43	40.1 ± 69.0	0.310	0.33	0.988	0.06

*^*a*^Median reported for rightward skewing data. Illinois-MT, Illinois modified test; SJ, squat jump; CMJ, countermovement jump; CMJA, countermovement jump with arms; 5JT, 5 jump test; RSTT, repeated sprint *T*-test; BT, best time; MT, mean time; TT, total time; FI, fatigue index; RL, right leg; A, anterior; BL, background left; BR, background right; LL, left leg.*

## Discussion

This study demonstrates that a 10-week in-season ULLPT program was effective in improving measures of upper body strength (handgrip force, back extensor, and medicine ball throwing), while permitting also substantial gains in sprinting, CoD, jumping, and RSTT performance in young female handball players, relative to participants who continued to follow the standard skills-based regimen. The findings confirm the observations of Hammami et al. (2019) who found improvements of handgrip force, back extensor strength, and medicine ball throwing in female handball players aged less than 14 years. Ignjatovic et al. (2012) also found increases in medicine ball throwing (*p* < 0.01), and in bench and shoulder press power (*p* < 0.05) relative to the controls after 12 weeks of medicine ball training in female handball players aged an average of 16.9 years (Ignjatovic et al., 2012), and Saeterbakken et al. (2011) reported a 4.9% increase in the maximal throwing velocity of female handball players aged 16.6 years after 6 weeks of medicine ball training (*p* < 0.01). Plyometric training improves strength through a combination of increased neural drive to the agonist muscles, improved intermuscular coordination, and changes in muscle size or architecture (Markovic and Mikulic, 2010). Increases in muscle strength and power after plyometric training could be due to neural and muscular adaptations such as changes in muscle architecture (i.e., a decrease in fascicle angle and an increase in fascicle length of knee extensors) (Blazevich et al., 2003); changes in the stiffness of various elastic components of the muscle-tendon complex of plantar flexors (Cornu et al., 1997; Kubo et al., 2007; Fouré et al., 2009; Grosset et al., 2009); and changes in single fiber mechanics of knee extensors (i.e., enhanced force, velocity and, consequently, power of slow, and fast muscle fibers) (Malisoux et al., 2007).

Plyometric training increases strength and power, with minimal effects on muscle volume (Markovic and Mikulic, 2010), so that gains in the upper limbs are unlikely to have negative consequences for sprinting ability. Hammami et al. (2019) previously found significant improvements in 20-m (*p* = 0.02, *d* = 0.557) and 30-m (*p* < 0.001, *d* = 1.07) performance after 9 weeks of ULLPT in U14 female handball players. Current results are consistent with these findings, with significant increases of speed over distances of 5–30 m. A meaningful portion of the ULLPT drills used during the current intervention implicated slow stretch-shortening cycle (SSC) muscle actions that mimicked those encountered during the acceleration phase of a sprint (Mero, 1988; Mero et al., 1992), contributing to the observed gains in sprint performance. Aside from the potential role of velocity-specific training, the direction of application of the muscle force may also contribute to the magnitude of the observed gains (Ramirez-Campillo et al., 2018). In this sense, the inclusion of horizontal jump drills may have contributed to the sprint improvements, given the importance of horizontal force production in sprinting (Morin et al., 2012; Kawamori et al., 2013). Improvement in sprint performance could be attributed to differences in the use of the SSC characteristics, as a SJ mainly consists of a concentric (push-off) phase, whereas a CMJ and other forms of plyometrics involve a coupling of eccentric and concentric phases (Markovic and Mikulic, 2010). The greatest benefits of plyometric training for sprint performance are dependent on the velocity of muscle action employed in training (Rimmer and Sleivert, 2000). Therefore, it has been suggested that greatest effects of PT on sprinting performance occur in the acceleration phase. It is known that slow SSC (long-response) plyometrics (>0.25 s), such as countermovement or SJs, transfer most directly to start and acceleration performance, whereas fast SSC (short-response) plyometrics (<0.25 s), such as drop jumps, have more transfer to maximum running velocity (Rimmer and Sleivert, 2000; Plisk et al., 2008).

The present results also showed a large effect group × time interaction in the Illinois-MT performance, matching the responses observed in the meta-analysis of Asadi et al. (2016) and Chaabene et al. (2019) but not Meszler and Vaczi (2019) (studies where training was limited to the lower limbs). Meszler and Vaczi (2019) explained their lack of response by fatigue, due to an incomplete recovery between sessions, but the regimen used also did not include any form of exercise where athletes had to change direction. Neural adaptations and enhancement of motor-unit recruitment may be mechanisms that can lead to an improvement on the CoD test (Aagaard et al., 2002).

A recent meta-analysis and other studies (Rubley et al., 2011; Chaabene et al., 2019; Meszler and Vaczi, 2019) have demonstrated increases in the jump performance of female athletes after plyometric training (Stojanovic et al., 2017), and the present results show that this benefit can be obtained if the regimen includes lower limb training. The improvement in jump performance is due to enhancing the elastic properties of the musculotendon unit and optimizing the neural sequencing and firing rates of the motor units involved (Ignjatovic et al., 2012). The increases observed in jump performance after ULLPT could be due to neuromuscular adaptations, such as an increased neural drive to the agonist muscles, changes in muscle-tendon mechanical-stiffness characteristics, alterations in muscle size and/or architecture, and changes in single-fiber mechanics (Maffiuletti et al., 2002; De Villarreal et al., 2009; Thomas et al., 2009). Other possible aspects of neural adaptation to plyometric training include (i) changes in leg muscle activation strategies (or inter-muscular coordination) during vertical jumping, particularly during the preparatory (i.e., pre-landing) jump phase; and (ii) changes in the stretch reflex excitability (Bishop and Spencer, 2004; De Villarreal et al., 2009).

This present study is the first to investigate the effect of plyometric training on repeated change of direction (RSTT) performance in young female handball players. It showed increases in all RSTT scores [best time (*p* < 0.001; *d* = 1.57); mean time (*p* < 0.001; *d* = 1.81); total time (*p* < 0.001; *d* = 1.81), and fatigue index (*p* = 0.49; *d* = 50)]. However, Rosas et al. (2017) previously noted increases in running anaerobic sprint test (RAST) after plyometric training in female soccer players, and (Hammami et al., 2016) found increases in 2 of 3 parameters (best time and total time) after 8 weeks of plyometric training in adolescent soccer players. Plyometric training affects nervous factors. It allows faster recruitment of motor units, a higher maximum discharge frequency, a greater quantity of innervation doublets, and better synchronization of motor units. All of the combined create a higher level of strength and a faster muscle contraction speed.

No significant gains in static or dynamic balance were seen after plyometric training. Others, also, have found no effects on balance from lower limb plyometrics alone. Thus, Hammami et al. (2019) found no effects on static and dynamic balance after 9 weeks of plyometric training in U14 female handball players, and Meszler and Vaczi (2019) saw no effects after 7 weeks of plyometric training in female basketball players aged less than 17 years, although Cherni et al. (2019) did report a significant improvement in the static balance test of adult female basketball players after plyometric training, and Benis et al. (2016) noted improved postural control and lower-limb stability as assessed by the *Y* balance test when adult female basketball players undertook body-weight neuromuscular training.

Certain limitations of this study must be acknowledged. The training load was not monitored over the training intervention using, for instance, external and/or internal load measures. If the group of players played an official match, then a friendly match or physical work was scheduled for the other group so that the load was the same for the two groups. Nevertheless, there is a difference in feeling between an official match, a friendly match and physical workload. This could potentially be checked by a mental trainer or by a psychology questionnaire, but unfortunately this is not the case in the current study. Notwithstanding, the general training description was quite detailed, with both groups following similar technical/tactical training over six training sessions per week, and we are confident that the total training load was similar between groups.

## Conclusion

This study demonstrated that the in-season combined ULLPT (two sessions per week) can substantially improve upper limb performance in young female handball players, while conserving the gains anticipated from lower limb plyometrics. The ULLPT intervention lasted a total of only 35 min per session, making it a time-efficient training method that can be routinely carried out in a sporting environment, with few resources required.

Upper and lower limb plyometric training is a time-efficient and a highly beneficial method for improving the physical performance of both upper and lower limbs in young female handball players. Further research is needed to examine the effects of ULLPT on muscle morphology and neural adaptations, and to explore the impact of maturation status as a potential moderator variable.

## Data Availability Statement

The raw data supporting the conclusions of this article will be made available by the authors, without undue reservation.

## Ethics Statement

The studies involving human participants were reviewed and approved by Manouba University Ethics Committee. Written informed consent to participate in this study was provided by the participants’ legal guardian/next of kin.

## Author Contributions

MH designed the study, conducted analyses, and wrote the manuscript. NG, RS, and KS assisted in acquisition, analysis and interpretation of data, and reviewed and edited the article. MH and MC administered the project. MC and KS made substantial contribution including conception and a critical revision of the article. All authors read and approved the final manuscript.

## Conflict of Interest

The authors declare that the research was conducted in the absence of any commercial or financial relationships that could be construed as a potential conflict of interest.
